# A common secretomic signature across epithelial cancers metastatic to the pleura supports IL-6 axis therapeutic targeting

**DOI:** 10.3389/fimmu.2024.1404373

**Published:** 2024-07-24

**Authors:** Vera S. Donnenberg, James D. Luketich, Bosko Popov, David L. Bartlett, Albert D. Donnenberg

**Affiliations:** ^1^ Department of Cardiothoracic Surgery, University of Pittsburgh School of Medicine, Pittsburgh, PA, United States; ^2^ UPMC Hillman Cancer Centers, Pittsburgh, PA, United States; ^3^ McGowan Institute for Regenerative Medicine, Pittsburgh, PA, United States; ^4^ Allegheny Health Network Cancer Institute, Pittsburgh, PA, United States; ^5^ College of Medicine, Drexel University, Philadelphia, PA, United States; ^6^ Department of Surgery, College of Medicine, Drexel University, Philadelphia, PA, United States; ^7^ Department of Medicine, College of Medicine, Drexel University, Philadelphia, PA, United States

**Keywords:** malignant pleural effusion, secretomics, tumor environment, epithelial to mesenchymal transition, IL-6 trans-signaling

## Abstract

**Background:**

Many cancers metastasize to the pleura, resulting in effusions that cause dyspnea and discomfort. Regardless of the tissue of origin, pleural malignancies are aggressive and uniformly fatal, with no treatment shown to prolong life. The pleural mesothelial monolayer is joined by tight junctions forming a contained bioreactor-like space, concentrating cytokines and chemokines secreted by the mesothelium, tumor, and infiltrating immune cells. This space represents a unique environment that profoundly influences tumor and immune cell behavior. Defining the pleural secretome is an important step in the rational development localized intrapleural immunotherapy.

**Method:**

We measured cytokine/chemokine content of 252 malignant pleural effusion (MPE) samples across multiple cancers using a 40-analyte panel and Luminex multiplexing technology.

**Results:**

Eleven analytes were consistently present in concentrations ≥ 10.0 pM: CXCL10/IP10 (geometric mean = 672.3 pM), CCL2/MCP1 (562.9 pM), sIL-6Rα (403.1 pM), IL-6 (137.6 pM), CXCL1/GRO (80.3 pM), TGFβ1 (76.8 pM), CCL22/MDC (54.8 pM), CXCL8/IL-8 (29.2 pM), CCL11/Eotaxin (12.6 pM), IL-10 (11.3 pM), and G-CSF (11.0 pM). All are capable of mediating chemotaxis, promotion of epithelial to mesenchymal transition, or immunosuppression, and many of are reportedly downstream of a pro-inflammatory cytokine cascade mediated by cytokine IL-6 and its soluble receptor.

**Conclusion:**

The data indicate high concentrations of several cytokines and chemokines across epithelial cancers metastatic to the pleura and support the contention that the pleural environment is the major factor responsible for the clinical course of MPE across cancer types. A sIL-6Rα to IL-6 molar ratio of 2.7 ensures that virtually all epithelial, immune and vascular endothelial cells in the pleural environment are affected by IL-6 signaling. The central role likely played by IL-6 in the pathogenesis of MPE argues in favor of a therapeutic approach targeting the IL-6/IL-6R axis.

## 1 Introduction

The most common adenocarcinomas to metastasize to the pleura are cancers of the lung, breast, pancreas, esophagus, and stomach (1). They manifest with pleural effusion and/or pleural thickening, nodules, or masses. Regardless of the site of origin, cancers metastatic to the pleura carry a dire prognosis compared to other metastatic sites. For example, median overall survival in metastatic hormone receptor positive (HR+) breast cancer (all sites) is 37 months (2). However, when HR+ breast cancer metastasizes to the pleura, it is uniformly fatal with a median survival of only 6 months (3). The prognosis is similar for patients with non-small cell lung cancer (median OS 6.3 months (4)) and other adenocarcinomas metastatic to the pleura.

We have argued that the principal reason for this uniformly poor prognosis across cancer types is that mutational profiles (5) and organ specific tropisms (6) are no longer the major factors determining outcome, being overshadowed by the potent effects of the pleural environment, which both suppresses anti-tumor immunity and promotes the epithelial to mesenchymal transition and aggressive tumor behavior (7, 8). In this report we extend our findings in non-small cell lung cancer and mesothelioma (9) and compare the secretomes of pleural effusions across a large dataset (n = 254 malignant pleural effusions) including multiple cancer types in order to understand their commonalities and differences, with the goal of identifying therapeutic targets to condition the maladaptive pleural environment and increase the efficacy of other therapeutic modalities (*e.g.*, immune checkpoint blockade, adoptive cellular therapy).

## 2 Materials and methods

### 2.1 Patients and samples

Pleural effusions (PE) were collected as anonymized medical waste under an IRB exemption (No. 0503126), or IRB approved protocol No. 16110093, under which patients consented to use of the sample and access to medical records. A total of 254 MPE were collected from patients with a variety of cancers (**Table 1**). Secretomic data for non-small cell lung cancer and mesothelioma have been previously published in part (9).

**Table 1:** Pleural effusion samples by cancer type.

Cancer Type	N
Breast	122
Esophageal	21
Non-small Cell Lung	61
Small Cell Lung	2
Melanoma	5
Mesothelioma	8
Ovarian	10
Renal	15
Other	20
**Total**	**254**

The category *Other* includes endometrial (1), colon (3), gastric (1), lymphoma (2), nasopharyngeal (1), pancreatic (2), plasmacytoma (2), prostate (1), urachal (1), urothelial (1) and origin unknown (5).

### 2.2 Secretomics

Secretomics were performed on MPE as previously described (9). Briefly, cells were removed by centrifugation (10 min at 600 x g, 4°C), and then further clarified (10 min 1880 x g, 4°C) prior to storage at -86°C. Immediately prior to analysis, samples were thawed and clarified by high-speed centrifugation (3 min at 16,000 x g, Beckman Microfuge E, Cat No. 348720, Beckman Coulter) in a coldroom environment (4°C).

A total of 40 cytokines and chemokines were quantified on the Luminex platform, using the Curiox LT-MX plate washer, Curiox DA-96 plates, the Luminex 200 System analyzer and xPonent data acquisition and analysis software. Standard curves were run for each cytokine with each sample batch. Cytokines were measured in 5 µL of neat, clarified effusion using the MILLIPLEX MAP Human Cytokine/Chemokine Magnetic Bead Panel - Premixed 38 Plex (Cat. No. HCYTMAG-60K-PX38), MILLIPLEX MAP Human TGFβ (Cat. No. TGFBMAG-64K-01), and IL-6Rα from the Human Angiogenesis/Growth Factor Panel 2 (Cat. No. HANG2MAG-12K-01). Determinations that were designated “Out of Range Below” (*i.e.*, below the limit of quantification) by the analytical software were arbitrarily filled with a value 1/10 the lowest valid measurement for that cytokine. Values designated “Out of Range Above” (*i.e.*, above the limit of quantification) were assigned the value of the highest valid measurement for that cytokine.

### 2.3 Statistical analysis

Because of the magnitude in size differences between cytokines and chemokines, results of the Luminex assay, reported in pg/mL, were converted to pM using molecular weights determined from the literature as reported previously in the Supplementary Material to reference (8). Molecular weights were not adjusted for glycosylation, isoforms or other variants. Secretomic analysis was performed on log_10_-transformed pM data. SYSTAT 13 software (San Jose, CA) was used for data analysis. Hierarchical clustering on columns was calculated using the one minus Pearson clustering metric in Morpheus (Broad Institute, https://software.broadinstitute.org/morpheus/). Discriminant analysis and ANOVA were performed in SYSTAT 13. The parameters for discriminant analysis were: backward stepwise estimation, tolerance = 0.001, F to enter = 4.0 and F to remove = 3.9. Coefficients of variation (cv) on log transformed data were calculated according to the method recommended by Canchola (10).

## 3 Results

### 3.1 Clustering between cancer types

**Figure 1:**
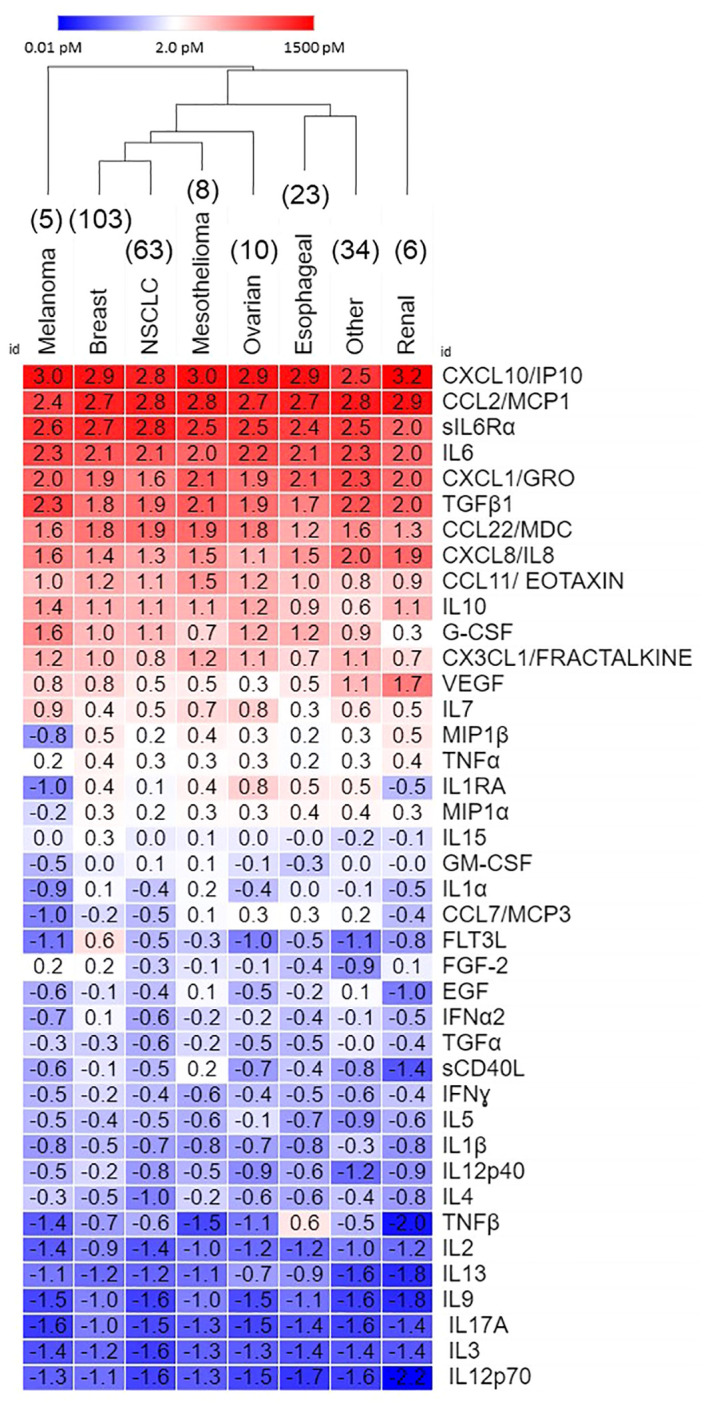
Secretome of malignant pleural effusions by disease. Data, expressed as log10 pM, are ordered on average concentration across all cancers. Hierarchical clustering was performed in Morpheus.

**Table 2** shows that eleven pleural cytokines and chemokines are significantly upregulated in the ≥ 10 pM range, and another eight in the 1 -10 pM range across epithelial cancers metastatic to the pleura. Hierarchical clustering between cancer types (**Figure 1**) showed breast cancer and non-small cell lung cancer, the diseases most frequently metastatic to the pleura, clustering together, and melanoma and renal cancers the farthest apart. Since the major cytokines and chemokines (**Figure 1**, red cells) were common across all cancer types, clustering was due to variability in analytes expressed at lower concentrations. TNFβ was most variable (cv = 176%), driven by high levels in esophageal cancer, followed by IL-1RA (high in ovarian cancer, low in melanoma and renal cancer, cv = 110%) and FLT3L (high in breast cancer, cv = 106%).

**Table 2:** Malignant pleural effusion geometric mean cytokine and chemokine levels across all cancers (n = 254), ordered by prevalence.

Analyte	Geometric Mean (pM)	LCI_95_	UCI_95_
CXCL10/IP10	672.28	586.73	770.29
CCL2/MCP1	562.86	504.69	627.74
sIL6Rα	403.09	337.99	480.74
IL6	137.58	118.72	159.43
CXCL1/GRO	80.25	67.42	95.52
TGFβ1	76.84	66.23	89.15
CCL22/MDC	54.83	46.91	64.08
CXCL8/IL8	29.17	23.64	35.99
CCL11/EOTAXIN	12.63	11.00	14.51
IL10	11.31	9.75	13.12
G-CSF	11.00	8.95	13.52
CX3CL1/FRACTALKINE	9.21	7.84	10.81
VEGF	5.02	3.66	6.89
IL7	3.08	2.58	3.67
MIP1β	2.18	1.76	2.71
TNFα	2.16	1.96	2.38
IL1RA	2.12	1.53	2.94
MIP1α	1.93	1.68	2.22
IL15	1.18	1.02	1.37
GM-CSF	0.96	0.79	1.17
IL1α	0.82	0.61	1.09
CCL7/MCP3	0.70	0.48	1.02
FLT3L	0.69	0.48	0.98
FGF-2	0.69	0.52	0.91
EGF	0.63	0.48	0.82
IFNα2	0.60	0.46	0.79
TGFα	0.46	0.37	0.55
sCD40L	0.45	0.34	0.59
IFNɣ	0.43	0.35	0.52
IL5	0.29	0.23	0.37
IL1β	0.27	0.23	0.33
IL12p40	0.27	0.20	0.36
IL4	0.24	0.18	0.32
TNFβ	0.24	0.15	0.39
IL2	0.08	0.06	0.09
IL13	0.06	0.05	0.09
IL9	0.05	0.04	0.07
IL17A	0.05	0.04	0.06
IL3	0.04	0.04	0.05
IL12p70	0.04	0.03	0.05

LCI_95_, lower 95% confidence interval; UCI_95_, upper 95% confidence interval.

### 3.2 Clustering between individual lung cancer samples

**Figure 2:**
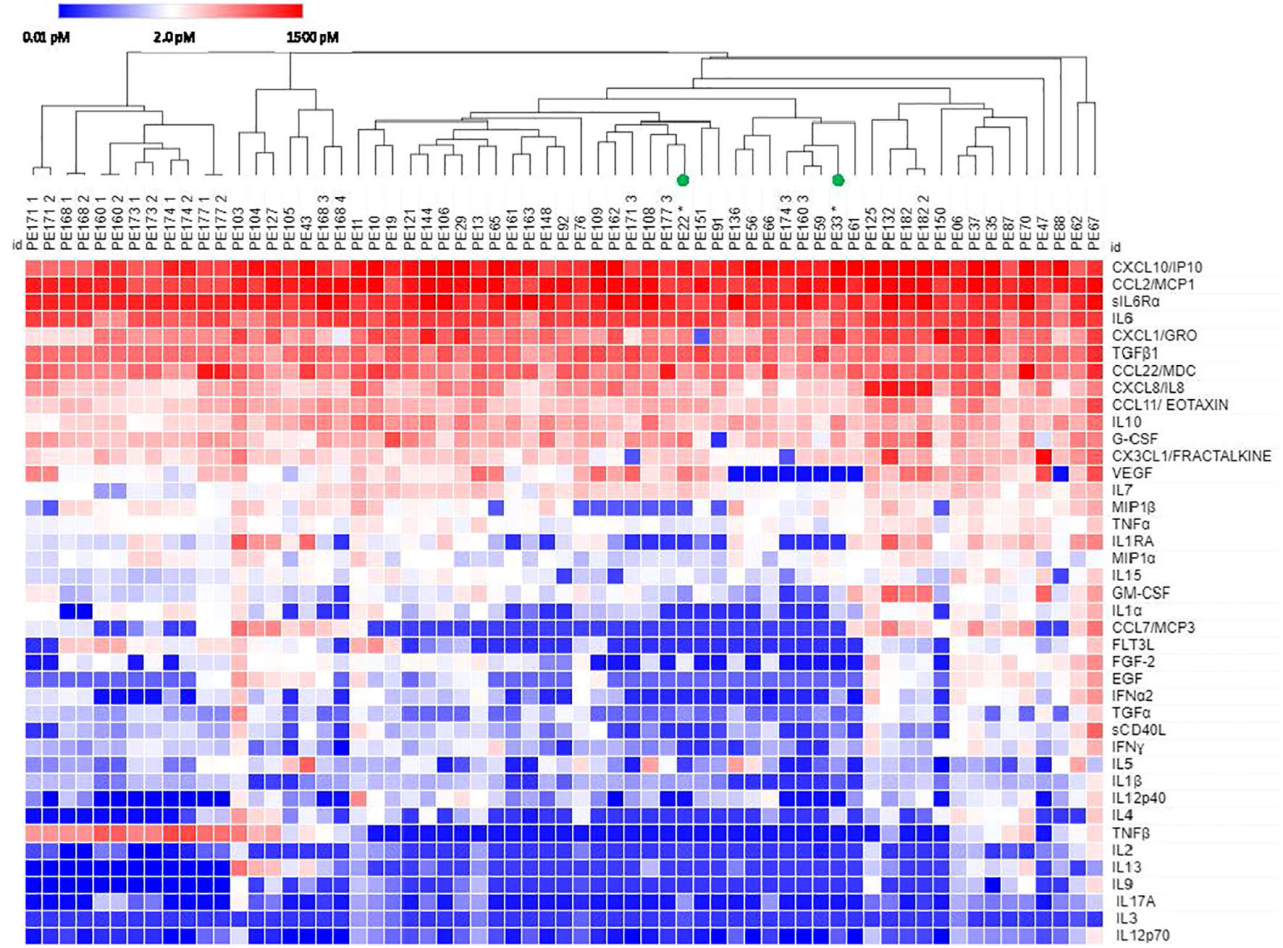
Hierarchical clustering of secretomic data in pleural effusions from lung cancer patients. Columns with the same PE number indicate repeat samples from the same patient. All patients had NSCLC except PE22 and PE33 (small cell lung cancer, green circles). Cytokines are ordered by prevalence in all cancers.

**Figure 2** shows inter-patient secretomic variability in patients with lung cancer metastatic to the pleura. We focused on lung cancer because it is a heterogeneous disease group, and the number of samples (n = 63) is amenable to graphic analysis. There was little variability among the cytokines and chemokines expressed at the highest concentrations (**Figure 1**). Seven patients had samples from effusions collected on different days, all of which clustered together, except for PE174, two of which clustered and one of which did not, due to lower levels of CX3CL1 and VEGF. As in all cancers (**Figure 1**), CX3CL1 (higher levels in melanoma and renal cancer), TNFβ, VEGF and IL-1RA showed the greatest variability of cytokines and chemokines expressed in the < 10.0 pM range.

### 3.3 Clustering between hormone receptor positive and triple negative breast cancer samples

**Figure 3:**
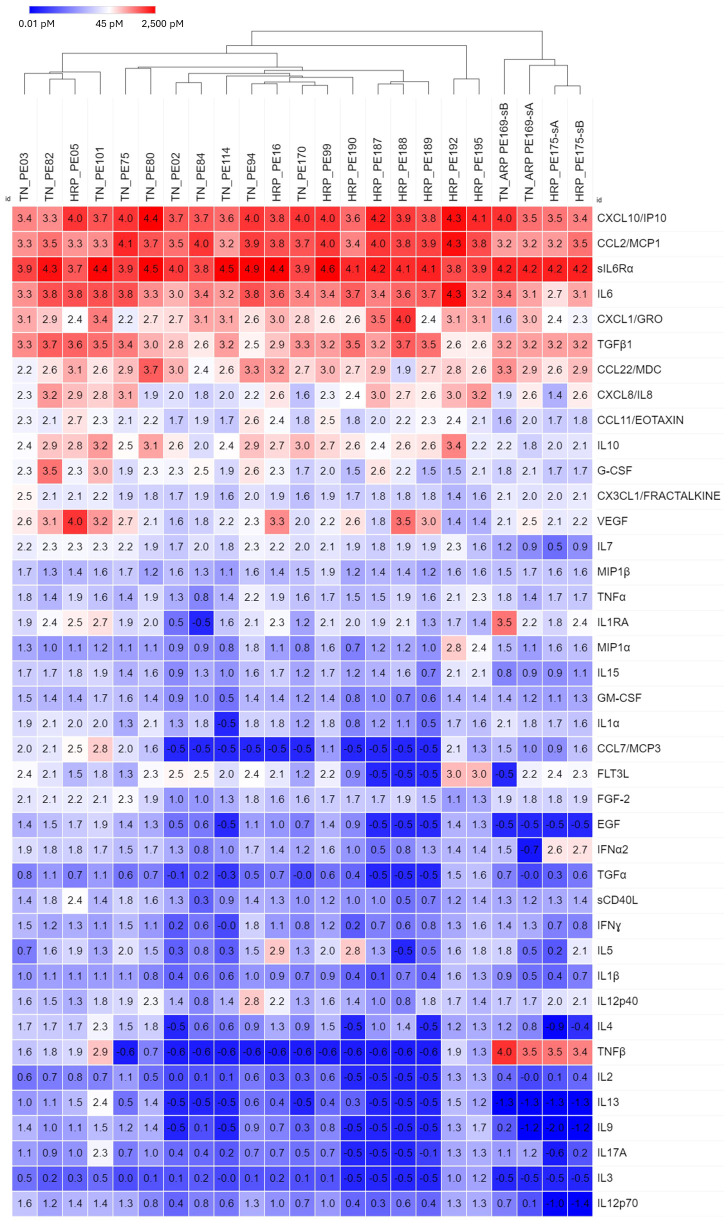
Hierarchical clustering of secretomic data in pleural effusions from breast cancer patients, grouped by hormone receptor status (HRP, hormone receptor positive; TN, Triple negative). In general, TN and HR+ samples tended to cluster, respectively, as did the 4 samples from *BRACA1+* patients with HR+ disease. Cytokines are ordered by prevalence in all cancers.

Of 122 breast cancer patients, 23 samples were collected from 21 patients who gave informed consent to obtain their medical records. These samples were classified by hormone receptor status (hormone receptor positive-all variants (HRP) *vs* triple negative (TN)). Even though all samples displayed high levels of the top 11 cytokines, samples tended to cluster by hormone receptor status (**Figure 3**). Four samples from two patients, one with TN/androgen receptor positive (ARP) disease, and one with HRP disease formed a separate cluster due to elevated TNFβ, low IL-7 and low EGF. No individual analyte was significantly different between hormone receptor groups (Student’s t-test, Bonferroni corrected).

### 3.4 Discriminant analysis distinguishes between breast cancer, lung cancer and esophageal cancer effusion secretomes

**Figure 4:**
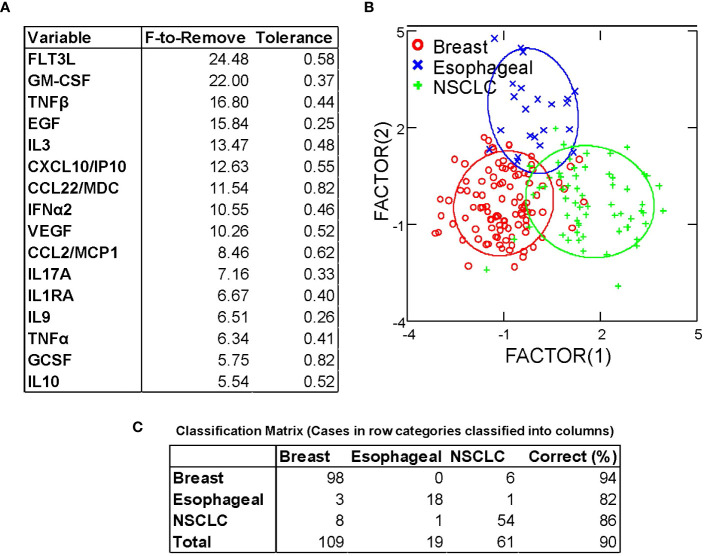
Discriminant analysis distinguishes between breast, esophageal, and non-small cell lung cancer based on cytokine/chemokine content of pleural effusion fluid. **(A)** Variables most influencing the ability to discriminate between cancer types, ordered by F-statistic. **(B)** Canonical Scores Plot. The axes are the first two canonical variables, a linear combination of the covariates (individual cytokine measurements) that provide maximum separation among the groups in 2 dimensions. The bivariate plot shows how each MPE sample is represented in terms of canonical variables and how each covariate contributes to the canonical variables. A 95% confidence level ellipse is plotted for the mean X,Y value of each disease group. If two groups differ significantly, the confidence ellipses tend not to intersect. **(C)** Classification matrix demonstrates the ability to predict disease type based on cytokine/chemokine levels.

Despite homogeneity in expression of the major chemokines and cytokines, the results of hierarchical clustering indicated consistent between-cancer differences (**Figure 1**). We performed discriminant analysis on breast cancer, non-small cell lung cancer, and esophageal cancer samples, the three largest groups in our dataset. These cancers were well-separated in the 40-dimensional space of the measured pleural secretome, with 89% of samples classifying correctly (**Figure 4**). This was confirmed by jackknifed resampling (83% classifying correctly). Cytokines most influential in separating the cancer types (F to remove ≥ 10) included FLT3L (highest in BrCA), GM-CSF (lowest in Eso), TNF-β (highest in Eso), EGF (lowest in NSCLC), IL-3 (lowest in NSCLC), CXCL10/IP10 (lowest in NSCLC), CCL22/MDC (lowest in Eso), IFNα2 (lowest in NSCLC, highest in BrCA), and VEGF (highest in BrCA), highest in BrCA). Other analytes that came up as significantly different between diagnoses in univariate statistics (ANOVA, Bonferroni corrected p-values) are: FGF-2 (highest in BrCA, p=0.01), IL-15 (highest in BrCA, p = 0.03), IL-12p40 (lowest in NSCLC, highest in BrCA, p = 0.03) and IL-2 (lowest in NSCLC, highest in BrCA, p = 0.02). The variability of these analytes may reflect disease-specific differences in immunogenicity or other tissue-specific attributes.

## 4 Discussion

### 4.1 Pleural space as a bioreactor

In malignant effusions and ascites, the cavitary space behaves much like a bioreactor. Because these cavities are lined by a mesothelial monolayer joined together with tight junctions (11), locally secreted cytokines and chemokines accumulate to high local concentrations in the absence of renal or hepatic clearance, the major mechanisms by which cytokines are eliminated from the peripheral circulation (12). Unlike solid tumors, in which the normal and malignant components interact within a tumor microenvironment, in MPE neoplastic and normal cells alike are bathed in a homogeneous macro-environment consisting of a rich mixture of cytokines and chemokines promoting and maintaining a mesenchymal tumor state (13) and rendering infiltrating immune effector cells unresponsive (7–9, 14).

### 4.2 Most prevalent cytokines and chemokines

Our data support this conclusion and show that 11 analytes are consistently elevated (≥ 10 pM) in the pleural fluid across a wide variety of cancer types. Of these, 6 are chemokines, small (8 – 14 kDa) peptide molecules that bind to G-protein coupled receptors. Chemokines were originally named for their chemotactic properties (a portmanteau of chemotactic and cytokine) and are responsible for the robust immune infiltrate that accompanies pleural effusions. The chemokine family of ligands has also been shown mediate a variety of other functions, playing a role in disease processes such as inflammation, autoimmunity, and cancer (15), and particularly in EMT and metastasis (16). We have previously shown that all the major chemokines identified here in MPE are constitutively secreted by cultured MPE tumor cells (8). CXCL10/IP-10 and CCL2/MCP1, the most abundant chemokines in our series are also secreted by cultured mesothelial cells, as is CXCL1/GRO (17). CXCL10 has been shown to synergize with TNF-α (also present in MPE) to induce EMT in colon cancer (18). MPE tumor-secreted CCL2 not only acts in an autocrine fashion to promote EMT and trans-endothelial migration, but also recruits tumor associated macrophages (19) and drives M2 polarization (20). MPE-tumor and mesothelial cells secrete CXCL1 (21, 22), CXCL8/IL-8 (23–25) and CCL11/Eotaxin (21, 26). All have been shown to promote EMT and tumor invasion, while macrophage-derived CCL22 (27, 28) drives alternative macrophage activation and IL-8 secretion (29) and recruits immunosuppressive cells to the tumor microenvironment (30).

### 4.3 The IL-6 axis

**Figure 5:**
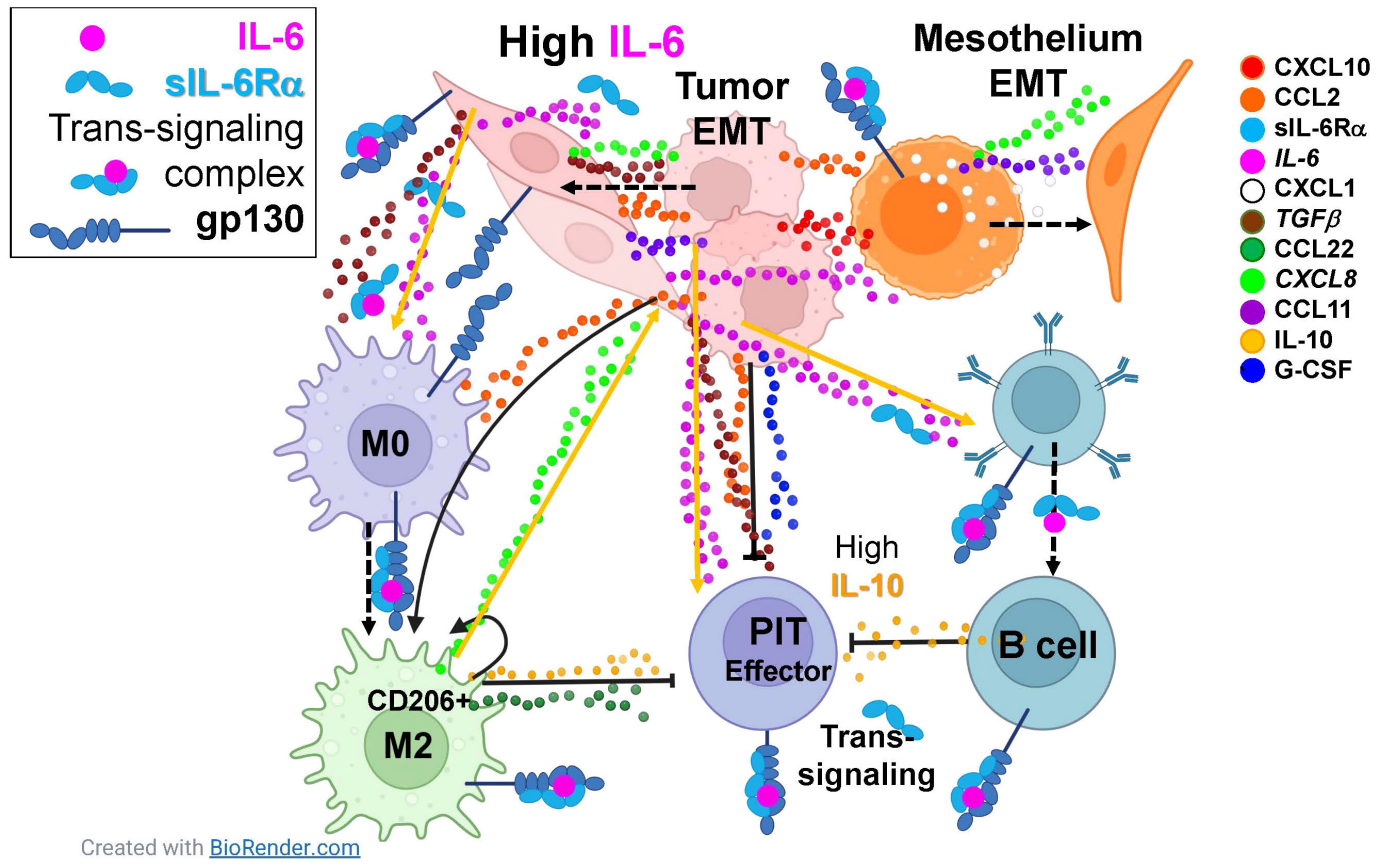
The IL-6 axis interacts with multiple cytokines to promote tumor and mesothelial EMT and suppress anti-tumor immunity. Eleven cytokines and chemokines were identified as consistently present in malignant pleural effusions with mean concentrations ≥ 10pM (legend on right). Dashed arrows indicate cellular phenotypic transitions; solid arrows indicate cytokine induced potentiation; capped lines indicate inhibition. All cell types in the pleura are responsive IL-6, constitutively secreted by the tumor and induced in mesothelial cells, either via classical or trans-signaling. Tumor and mesothelial cells are driven to the epithelial to mesenchymal transition (EMT) by paracrine and autocrine CXCL10, CCL2, IL-6, CXCL1, TGFβ, CXCL8, and CCL11. Macrophages are driven to an M2 state by CCL2, IL-6, and TGFβ. Although effector pleural infiltrating T cells (PIT) can be stimulated by IL-6, they are suppressed by CCL2, IL-10, G-CSF, TGFβ and CCL22. Pleural infiltrating B cells are driven by IL-6 to secrete the immunosuppressive cytokine IL-10. Taken together, the natural immune environment of the pleural cavity, in combination with IL-6-mediated wound-healing signals provided by the tumor, synergize in a maladaptive response that promotes aggressive tumor behavior and suppression of anti-tumor effector responses.

The most prominent cytokines in our series, common to all cancers, are IL-6 and its soluble receptor IL-6Rα, TGFβ, IL-10, and G-CSF. The pleiotropic master cytokine IL-6, when bound to the soluble alpha-chain of its receptor, is capable of trans-signaling to the wide variety of cells that express gp130, the IL-6 signal transducing protein. Given the right stimuli, IL-6 secretion can be induced in immune and stromal cells, but cultured MPE tumor cells secrete it constitutively (8). The IL-6/IL-6Rα complex initiates pathologic cytokine cascades in many pro-inflammatory disease states (31) and stimulates production of the immunosuppressive cytokines IL-1RA and IL-10 (32), both present in MPE. IL-6 and TGFβ are also potent inducers of EMT (13, 33). Further, IL-6 increases membrane trafficking of TGFβ receptor, augmenting TGFβ signaling (34) and regulatory B-cell differentiation (35). TGFβ (36) and G-CSF (37), both physiologic enforcers of immunologic tolerance, are secreted by MPE tumor cells (8) and contribute to the immunosuppressive pleural environment. TGFβ also promotes an M2 macrophage polarization (38). The relationship between IL-6 and G-CSF is complex, but co-stimulation with IL-6 and G-CSF is reported to induce protumor function in neutrophils (39), and IL-6/IL6Rα trans-signaling promotes G-CSF-independent granulopoiesis upon exposure to pathogens (40). IL-10 is a pleiotropic cytokine shown to downregulate expression of Th1 cytokines (41), MHC class II antigens (42), and co-stimulatory molecules on macrophages (43). Its expression is enhanced by IL-6 (32). IL-6 trans-signaling also promotes secretion of CXLC8 and CCL2 by rheumatoid arthritis synoviocytes, a cell type related to mesothelial cells (44). In our series, the geometric mean molar ratio of sIL-6Rα to IL-6 was 2.7 (LCI_95_ = 2.1, UCI_95_ = 3.3), greatly facilitating trans-signaling within the pleural environment and ensuring that gp130+ normal and malignant epithelial cells, immune cells and vascular endothelial cells in the pleural environment are responsive to IL-6 signaling. **Figure 5** shows, in schematic, how the IL-6 axis may interact with multiple cytokines to promote tumor and mesothelial EMT and suppress anti-tumor immunity.

One mechanism that may explain the relationship between IL-6 and the pathophysiology of benign and malignant pleural effusions is the association of specific IL-6 and IL-6R polymorphisms with altered inflammatory responses (45). Subjects with SNPs associated with decreased IL-6 classical signaling have lower odds of tuberculosis disease (46). Conversely, polymorphisms resulting in elevated IL-6 levels or signaling have greater risk of allergy (47), proliferative diabetic retinopathy (48) and other immune-related pathologies (45). It remains to be determined whether specific IL-6 or IL-6R polymorphisms predispose to effusions and/or intracavitary metastasis.

### 4.4 Within- and between-disease variation in effusion cytokine content

The within-disease variation in cytokine content for a single, but heterogeneous disease site (lung cancer, **Figure 2**) is similar to the variation between diseases (**Figure 1**), demonstrating the consistency with which the major cytokines and chemokines comprise the pleural secretome across different cancers.

Despite the major secretomic commonalities between cancers metastatic to the pleura, differences in tissue of origin contributed to subtle but consistent between-disease differences in cytokine and chemokine content. When we used discriminant analysis to compare breast, lung and esophageal cancers, the most prevalent cancers in our dataset, their secretomic profiles easily distinguished between the diseases (**Figure 4**). Many of the most influential cytokines were present at low concentration. Several were associated with effector responses such as IFNα, TNFα, TNFβ, and IL-9, possibly reflecting inherent disease-specific differences in immunogenicity.

## 5 Conclusions

The most salient finding in this report is that the major cytokines conditioning malignant pleural effusions and contributing to their aggressive pathobiology are the same across cancers. Even across cancers with disparate biologies and etiologies, the cavitary environment can have such a profound effect as to make them equally aggressive, equally immunosuppressive, and equally therapy resistant. Cytokines present at lower levels allowed breast cancer patients to cluster by hormone receptor status (**Figure 3**), and distinguish between patients with breast, lung and esophageal cancers (**Figure 4**).

The present data support the contention IL-6 signaling is upstream of a maladaptive cytokine and chemokine cascade capable of suppressing anti-tumor effector responses at the level of effector T cells and macrophages and promoting EMT and aggressive tumor behavior. Since IL-6 is also elevated in both transudative and exudative benign effusions (9), there is the distinct possibility that IL-6 is the major driver of all effusions, once initiated by mechanical or inflammatory stimuli (49, 50). There is abundant precedent for targeting IL-6 or its soluble receptor to ablate pathologic cytokine release cascades associated with rheumatoid arthritis (51), adoptive immunotherapy (52), or infectious disease and sepsis (53, 54). Importantly, such therapy does not appear to interfere with anti-tumor immune effector responses (52), raising the possibility that localized therapy directed against the IL-6 axis could counter EMT while unleashing a preexisting, but silenced (8) local anti-tumor response.

The risks of intra-cavitary administration of drugs targeting the IL-6 axis are presently unknown. In clinical trials evaluating intravenous administration of single agent tocilizumab in patients with rheumatoid arthritis, infection was the most common serious adverse event, in keeping with its anti-inflammatory effects (55). The most common adverse reactions were upper respiratory tract infections, nasopharyngitis, headache, hypertension and increased alanine transaminase. Twenty-four percent of patients required dose modification or interruption due to adverse events. We hypothesize that such toxicities can be minimized by intracavitary administration, attaining high local drug concentration with significantly lower total doses, with minimal systemic spillover. We are currently conducting an open label Phase I dose escalation trial of intracavitary tocilizumab in patients with malignant pleural effusions and ascites (NCT06016179) (56).

The implications of the present study are threefold: 1) The prominence of IL-6 and IL-6Rα suggest a therapeutic strategy. Antibodies directed against IL-6 or its soluble receptor may counter both EMT and immunosuppression, and may even ameliorate the effusion itself; 2) Therapeutic agents can be delivered directly to the pleural cavity to counteract the maladaptive pleural environment and facilitate natural or adoptive immune effector responses against the tumor without incurring systemic toxicity (7, 8); 3) This strategy could be employed across all cancers that are metastatic to the pleura, providing that the mutational burden is sufficient to generate immunogenic peptides against which anti-tumor responses can be directed. Between-patient differences in low level effector cytokines may provide a clue as to who will respond to localized immunotherapy.

Taken together, the data indicate a strong commonality between diverse cancer types, not only in IL-6, but in the major cytokines and chemokines comprising the pleural secretomic milieu. The results identify the IL-6/IL-6Rα/gp130 signaling complex, which affects virtually every cell in the pleural environment, and which can provoke a pathologic cascade of cytokines, as a potential therapeutic target.

## Data availability statement

The raw data supporting the conclusions of this article will be made available by the authors, without undue reservation.

## Ethics statement

The studies involving humans were approved by University of Pittsburgh Human Research Protection Office. The studies were conducted in accordance with the local legislation and institutional requirements. The participants provided their written informed consent to participate in this study.

## Author contributions

VD: Conceptualization, Formal analysis, Investigation, Supervision, Writing – original draft, Writing – review & editing, Funding acquisition. JL: Resources, Validation, Writing – review & editing. BP: Methodology, Writing – review & editing. DB: Resources, Writing – review & editing. AD: Conceptualization, Formal analysis, Investigation, Supervision, Writing – original draft, Writing – review & editing.
